# Impact of Dietary Protein and Gender on Food Reinforcement

**DOI:** 10.3390/nu9090957

**Published:** 2017-08-30

**Authors:** Shanon L. Casperson, James N. Roemmich

**Affiliations:** United States Department of Agriculture, Agricultural Research Service, Grand Forks Human Nutrition Research Center, 2420 2nd Ave. North, Grand Forks, ND 58203-9034, USA

**Keywords:** food reinforcement, protein, snack foods, energy-dense foods, motivated behavior, eating behavior

## Abstract

Recent evidence suggests that increasing dietary protein may alter reward-driven eating behavior. However, the link between protein and food reinforcement is not known. We sought to determine the extent to which increasing dietary protein alters food reinforcement in healthy adults. In a randomized crossover study, 11 women (age = 25 ± 7 years; Body Mass Index (BMI) = 21 ± 2 kg/m^2^) and 10 men (age = 22 ± 2 years; BMI = 24 ± 2 kg/m^2^) consumed normal (15%) and high (30%) protein meals. Food reinforcement was assessed using a computer-based choice task (operant responding with concurrent log_2_(x) reinforcement schedules) 4 h after lunch. We found that food reinforcement was greater in men than women (*p* < 0.05) and greater for sweet than savory snack foods (*p* < 0.02). Gender interacted with dietary protein level (*p* = 0.03) and snack food type (*p* < 0.0001). Specifically, we found that increasing dietary protein decreased the reinforcing value of savory foods in women. The reinforcing value for sweet foods did not interact with dietary protein or gender. These results demonstrate the differential effects of dietary protein on the reinforcing value for energy-dense, highly palatable snack foods.

## 1. Introduction

The role of dietary protein on increasing satiation, satiety, and energy metabolism is well documented [1]. However, eating behavior is modulated by not just meal or diet macronutrient composition, but also the interactions among physiological need (homeostatic), the rewarding properties of food, and our environments [2]. Changes to our food environment, coupled with the increase in adiposity over the last four decades, suggest that eating behavior is influenced more by nonhomeostatic contributors than homeostatic regulatory mechanisms. With the seemingly constant availability of energy-dense, highly palatable (enjoyable and appetizing) foods, understanding eating behavior beyond the physical need has become a priority [2]. One factor that influences eating behavior is the reinforcing value of the foods available in an environment [3]. Moreover, food reinforcement is positively associated with obesity [4]. The reinforcing value of a food is a learned response that is associated with that food’s reinforcement history (positive or negative) and has been shown to be modifiable depending upon baseline intake and the availability of the alternative choices [4,5]. Therefore, elucidating the motivating properties of food is essential in developing successful strategies that work to enhance homeostatic regulation of food intake.

Dietary protein has been posited to influence food reinforcement regulatory mechanisms. Data from animal and human magnetic resonance imaging (MRI) studies demonstrate that greater protein intake diminishes activation of central motivation and reward areas of the brain [6]. In animals, intragastric infusion of physiological amounts of protein decreased stimulation in the amygdala compared to a mixed nutrient meal or intravenous glucose infusion [7]. In humans, greater dietary protein reduces activation in the mesocorticolimbic circuitry (notably the insular cortex, prefrontal cortex, hippocampus, and parahippocampus) [8,9,10]. Collectively, these results demonstrate the potential role of dietary protein in modulating food reinforcement. However, it is not clear if reduced activity in these reward areas of the brain translates into actual decreases in reward-driven eating behaviors. 

Food reinforcement has been studied using a computer-based choice task (operant responding) in a variety of settings and is measured by the amount of operant responding (i.e., work) an individual will complete to earn a small portion of the food [3]. If the food is highly reinforcing, it will support more work to earn access to that food [4]. The present study extends previous research by employing such an operant responding task to test the effects of increased dietary protein on actual motivated behavior and the reinforcing value of foods with specific taste profiles in healthy weight adults. We hypothesized that increasing dietary protein would decrease the reinforcing value for energy-dense, highly palatable snack foods.

## 2. Materials and Methods

### 2.1. Participants

Healthy weight women (*n* = 11; age = 25 ± 2 years; Body Mass Index (BMI) = 21 ± 1 kg/m^2^) and men (*n* = 10; age = 22 ± 1 years; BMI = 24 ± 0.5 kg/m^2^) were recruited from the Greater Grand Forks area through the Grand Forks Human Nutrition Research Center (GFHNRC) website and flyers posted in local businesses for participation in the study. The study was approved by the University of North Dakota Institutional Review Board and registered at http://clinicaltrials.gov (NCT02211599). Informed written consent was obtained prior to participation. Participant eligibility was determined during a screening exam at the GFHNRC. Exclusion criteria included: BMI <18 and > 25kg/m^2^, allergies to any study foods, recent weight loss or gain, pregnancy, lactation, fasting glucose >100 mg/dL, active cancer or in short-term remission (less than three years), infectious diseases, alcohol or drug abuse, tobacco use, presence of acute illness, or taking medications known to affect appetite. Participants were instructed to continue all regular activities of daily living and maintain their usual diet.

### 2.2. Experimental Procedures

Participants completed a randomized cross-over study at the GFHNRC to determine the impact of dietary protein (15% of energy intake (E) vs. 30% E) and gender on the reinforcing value of energy-dense snack foods. Study visits were separated by a minimum of one week. Women were scheduled on the same day of their menstrual cycle to control for possible confounding effects on food cravings. Participants completed a three-day food diary prior to each study visit to determine habitual macronutrient intakes.

Participants reported to the GFHNRC Metabolic Research Unit the evening before each testing day. All meals were prepared and weighed by the GFHNRC research kitchen. A standardized meal (57% carbohydrate, 15% protein, and 28% fat), to control for nutrient intake and timing, was provided at 19:00 h. The breakfast (08:00 h) and lunch (12:00 h) test meals were comprised of the same foods; however, the meals were presented differently at each meal to minimize monotony (Table 1). Each meal provided 500 calories; fat intake was set at 30% and carbohydrate intake was adjusted to maintain isocaloric conditions. Absolute protein intake was 19 g and 38 g per meal for the 15% E and 30% E protein diets, respectively. Participants were required to consume all foods.

Indices of appetite sensations were measured immediately prior to the food reinforcement task using a computer-based visual analogue scale [5]. The reinforcing value for sweet and savory snack foods was assessed 4 h after lunch using a computer choice task as previously described [5]. Briefly, two separate computer workstations were used to simulate a behavioral choice between energy-dense, highly palatable sweet and savory snack foods. On one computer, the participant played a slot machine like game to earn points for a small portion (70–100 kcal) of their most liked sweet snack food. On the other computer, the participant played the same type of game to earn points for the same size portion of their most liked savory snack food (Table 2). Participants worked at their pace and could freely distribute their operant responding to the schedules. The behavioral cost to gain access to each snack food increased on independent and concurrent log_2_(x) reinforcement schedules. The reinforcement schedule used for this study was 4, 8, 16, 32, (…), 1024 mouse clicks for each point. Participants received one reward each time five points was earned. The schedule of reinforcement advanced after the reward was earned. Participants’ most-liked snack food choices used as the food rewards were determined prior to start of study.

### 2.3. Food Reinforcement Task

The total number of mouse clicks (operant responding; P_response_) and the breakpoint (P_max_, last schedule completed) were recorded for each snack food. The reinforcing value of the sweet snack food relative to the savory snack food (RRV_sugar_) was calculated as

RRV_sugar_ = P_max for sweet_/(P_max for sweet_ + P_max for salty/savory_). (1)

As calculated, an RRV_sugar_ greater than 0.5 indicates a greater reinforcing value of the sweet snack food relative to the savory snack food. Total responses, breakpoint and the RRV were used as metrics of food reinforcement [3,5].

### 2.4. Statistical Analysis

P_response_ and P_max_ were analyzed using a mixed model analysis of variance with gender (F or M), dietary protein (15% E or 30% E) and snack preference (sweet or savory) and their interactions included as fixed effects and participants as random effects. A logarithmic transformation of the P_response_ data was used to reduce skewness. The effects of gender and dietary protein on appetite sensations and the RRV of sweet to savory snack foods was assessed using a mixed model analysis of variance in which gender and dietary protein were fixed effects and the participant was treated as a random effect. SAS V9.4 (SAS Institute, Inc., Cary, NC, USA) was used for all analyses. P_response_ is presented as the back-transformed mean (−1SE, +1SE). All other data are reported as means ± standard error (SE). Power analysis, based on the first 5 participants, indicated that 10 participants per group provided 90% power to detect a protein and gender effect of 0.30 for RRV of food given a within-subject standard deviation of 0.21; *p* = 0.05. 

## 3. Results

### 3.1. Reinforcing Value of Snack Foods

**Figure 1:**
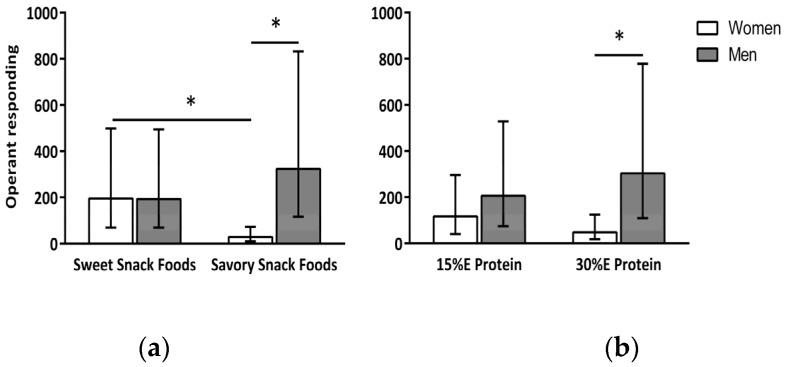
Interaction between gender and snack food type (**a**) and between gender and dietary protein (**b**) on snack food reinforcement. Data are expressed as back-transformed means (−1SE, +1SE). * indicates a significant (*p* < 0.05) difference between means. As shown in (**a**), reinforcing value as measured by operant responding was greater for sweet than savory snack foods for the women only. Reinforcing value of savory snack foods was greater in men than women, but, there was no gender difference in the reinforcing value of sweet snack foods. As shown in (**b**), when the meal contained 30% E protein women’s reinforcing value of snack foods was lower than that of the men.

A main effect of gender (*F*(1,19) = 4.49, *p* < 0.05) and snack food type (*F*(1,53) = 5.78, *p* < 0.02) was found for P_response_. Operant responding (P_response_) for snack food was greater in the men (250 (167, 374) mouse clicks) compared to the women (75 (50, 111) mouse clicks) and for the sweet (194 (141, 266) mouse clicks) compared to the savory (97 (71, 133) mouse clicks) snack foods. In addition, there was a significant interaction between gender and snack food type, *F*(1,53) = 17.75, *p* < 0.0001 (Figure 1a). The women’s P_response_ was greater for the sweet (195 (125, 303) mouse clicks) compared to the savory (29 (18, 44) mouse clicks) snack foods (*p* = 0.0001). For the men, there was no difference in P_response_ for the sweet (193 (123, 302) mouse clicks) and savory (324 (206, 508) mouse clicks) snack foods. There was also a significant interaction between gender and dietary protein level, *F*(1,53) = 4.69, *p* = 0.03 (Figure 1b). When the meal contained 15% E protein there was no difference in P_response_ between the women (117 (76, 180) mouse clicks) and men (206 (131, 323) mouse clicks). However, when the meal contained 30% E protein the women’s P_response_ was markedly less (*p* < 0.03) than that of the men (48 (30, 76) and 303 (193, 475) mouse clicks, respectively). 

**Figure 2:**
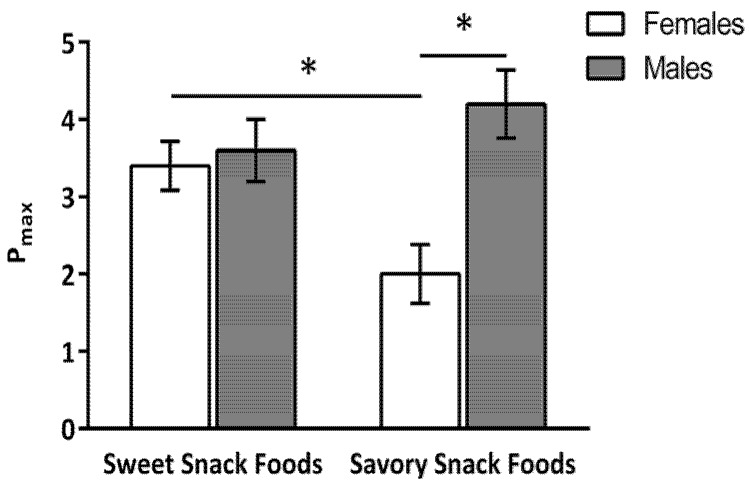
Interaction between gender and snack food type for Pmax (number of schedules completed to earn a snack food reinforcer). * indicates a significant (*p* < 0.05) difference between means. Data are expressed as mean ± standard error (SE). P_max_ for sweet snack foods was greater than for the savory snack foods for the women only. P_max_ for the savory snack foods was greater in men than women but, there was no gender difference in the P_max_ of the sweet snack foods.

No significant main effect of gender, snack food type, or dietary protein level was found for P_max_. However, there was a significant interaction between gender and snack food type, *F*(1,53) = 14.68, *p* < 0.01 (Figure 2). For the women, P_max_ was greater for the sweet (3.4 ± 0.3 schedules) compared to the savory snack foods (2.0 ± 0.4 schedules). For the men, there was no difference in the P_max_ for the sweet (3.6 ± 0.4 schedules) and savory (4.2 ± 0.4 schedules) snack foods. Additionally, P_max_ for the savory snack foods was greater in the men than the women (*p* < 0.02). There was no difference in P_max_ between the women and men for the sweet snack foods.

**Figure 3:**
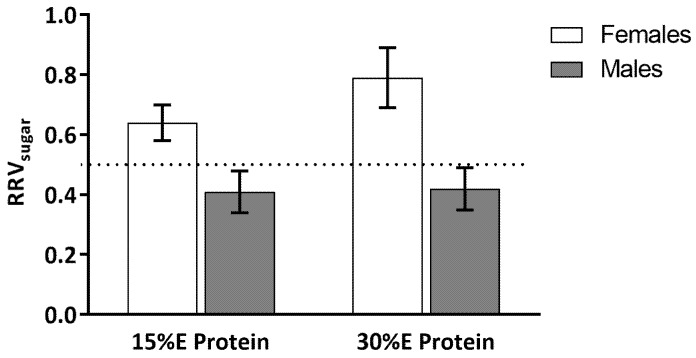
Relative reinforcing value (RRV_sugar_) of sweet to savory snack food. There were significant main effects of gender (greater in women) and dietary protein (greater after 30% E protein meal) on RRV_sugar_. Values are expressed as means ± SE.

A main effect of gender (*F*(1,18) = 13.17, *p* < 0.01) and dietary protein level (*F*(1,17) = 4.48, *p* = 0.05) was found for RRV_sugar_ (Figure 3). The interaction between gender and dietary protein level did not reach significance, *F*(1,17) = 3.48, *p* = 0.08. For the women, when the meal contained 15% protein the RRV_sugar_ was 0.59 ± 0.05 and when the meal contained 30% E protein RRV_sugar_ increased to 0.78 ± 0.09. This increase in RRV_sugar_ was driven by a decrease in the reinforcing value of the savory snack food (see Figure 2). For the men, when the meal contained 15% E protein, RRV_sugar_ was 0.45 ± 0.03 and when the meal contained 30% protein the RRV_sugar_ was 0.47 ± 0.03. 

### 3.2. Appetite Sensations

Main effects of gender and dietary protein level were found for hunger (*F*(1,30) = −16.40, *p* < 0.01 and *F*(1,18) = 5.18, *p* = 0.04, respectively) and fullness (*F*(1,30) = 12.79, *p* = 0.01 and *F*(1,18) = 5.54, *p* = 0.03, respectively). Women reported feeling less hungry and fuller than the men. Increasing dietary protein decreased feelings of hunger and increased feelings of fullness. There was no interaction between gender and dietary protein level.

A main effect of gender was found for the desire to eat something savory (*F*(1,35) = 13.91, *p* < 0.01). The desire to eat something savory was less in the women than the men irrespective of dietary protein level. The desire to eat something sweet did not differ between the women and men and was not influenced by dietary protein level.

### 3.3. Snack Food Intake

A main effect of gender was found for the amount of savory snack foods consumed, *F*(1,19) = 10.55, *p* < 0.01. The caloric consumption of savory snack foods was less in the women (107 ± 20 kcal) than the men (275 ± 33 kcal), irrespective of dietary protein level. Conversely, there were no significant main effects, nor an interaction between, gender and dietary protein level for the amount of sweet snack foods consumed. RRV_sugar_ predicted the amount of savory snack foods (*F*(1,18) = 30.14, *p* < 0.01), but not the amount of sweet snack foods (*F*(1,18) = 1.47, *p* = 0.24), consumed by each participant.

### 3.4. Habitual Dietary Intake

Habitual dietary intake did not differ prior to each study visit. Average energy intake for the women was 1993 ± 103 kcal/day with a macronutrient composition of 48 ± 2% E carbohydrates, 16 ± 1% E protein, and 35 ± 1% E fat. Habitual protein consumption was 77 ± 5 g protein/day or approximately 1.3 ± 0.1 g protein/kg/day. Daily energy intake for the men was 2240 ± 128 kcal/day with a macronutrient composition of 41 ± 1% E carbohydrates, 21 ± 1% E protein, and 37 ± 2% E fat. Habitual protein consumption was 113 ± 8 g protein/day or approximately 1.5 ± 0.1 g protein/kg/day.

## 4. Discussion

The present study was conducted to examine the effect of normal (15% E) and high-protein (30% E) meals on the reinforcing values of energy-dense, highly palatable sweet and savory snack foods in healthy weight adults. To our knowledge, this is the first study to test the effect of protein intake on the amount of work an individual is willing to perform to gain access to a highly reinforcing food. Our results show that increasing dietary protein differentially altered food reinforcement in women and men. Most notably, increasing dietary protein intake decreased the reinforcing value of savory foods in women. 

The present study is the first to demonstrate an effect of protein intake on the motivation to gain access to a highly rewarding food, as assessed by operant (behavioral) responding, and eating behavior. By demonstrating an effect of dietary protein on behavioral responses, it extends recent neuroimaging studies that have shown that increasing protein intake decreases activation in central motivation and reward areas of the brain; potentially decreasing reward-driven eating [8,9,10]. In overweight/obese late-adolescent girls, a high protein breakfast reduced brain activation responses to visual food cues in the anterior insular and mid-prefrontal cortex 3 h postprandial (pre-lunch) [8], and in the hippocampus and parahippocampus regions 8 h after breakfast (pre-supper) [9] compared to a normal protein breakfast (40% E vs. 15% E, respectively). The reduced activation of the aforementioned brain regions coincided with a decrease in nighttime snacking on high-fat foods [9]. Furthermore, feeding healthy weight women a low-protein (7% E) or high-protein (25% E) diet for 16 days resulted in contrasting postprandial changes in reward-related areas of the brain [10]. Specifically, increasing dietary protein resulted in decreased activation in the inferior orbitofrontal cortex in response to savory food cues [10]. Taken together, the results from these neuroimaging studies indicate that dietary protein may modulate reward-driven eating behavior in females. The findings of the current study expand upon the knowledge gained from these neuroimaging studies by demonstrating a significant interaction between dietary protein and gender on motivated behavior for energy-dense savory snack foods. Thus, in women, increasing protein intake modifies eating behavior, specifically, the consumption of savory foods. 

The current results indicate that increasing dietary protein from 15% E to 30% E does not alter motivated behavior in men. These findings are supported by those of Frank et al. [11] who found that changes in neural responses in brain regions responsible for reward-seeking behavior were only visible in women, but not in the men [11]. Furthermore, Sayer and colleagues [12] reported no differences in postprandial neural responses to visual food cues 3 h after consuming normal (12 g) and high-protein (25 g) breakfasts in overweight adults (6 women and 12 men). The disproportionate number of women and men in their participant pool may have led to their results more closely resembling the responses that the current and previous study [11] detailed for men. Taken together, these findings suggest that gender plays an important role in the neurobehavioral responses to dietary protein. Further investigation is necessary to continue to elucidate the effects of gender on eating behavior in response to alterations in dietary macronutrient composition.

Interestingly, increasing dietary protein from 15% E to 30% E did not alter the reinforcing value of sweet foods. This unexpected finding opposes our hypothesis that the reinforcing value of, and consumption of, sweet foods would be lessened after consuming meals containing 30% E compared to 15% E protein. Previous research had shown that increasing dietary protein from 15% E to 40% E [9] or from 10% E to 25% E [13] decreases snacking on energy-dense sweet foods. On the other hand, Gosby et al. [13] reported no measurable differences in the intake of sweet foods when dietary protein was increased from 15% E to 25% E. Therefore, it can be postulated that an increase of at least 15% E, or an increase from a relatively low baseline protein intake of 10% E, is needed to elicit a reduction in the consumption of sweet foods. However, we found no effect of a 15% E increase in dietary protein on the intake of sweet foods. Therefore, a larger divergence between habitual and meal protein intake might be needed in order to alter motivated behavior for sweet foods. Future studies should systematically explore the possibility of a threshold at which motivated behavior is increased or decreased in response to changes in protein intake.

This study is not without limitations. First, only healthy weight adults were recruited for participation. Weight status influences the RRV of food and energy intake [4], therefore, it is possible that overweight and obese individuals may exhibit different appetitive or motivational responses in response to changes in dietary protein. However, the current study of healthy weight adults provides an initial indication of how dietary protein can alter motivation for, and consumption of, energy-dense sweet and savory snack foods later in the day. Second, only a limited number of snack foods were studied. It is possible that the RRV of snack foods with different taste profiles than those tested here would have produced different results. Still, it is intriguing that increasing dietary protein decreased the RRV of foods with a savory taste profile in the women only. 

## 5. Conclusions

Eating behavior is driven by the complex interrelationship between homeostatic and nonhomeostatic inputs. One implication of this research is that, for women, modulation of nonhoemostatic regulation of eating behavior by dietary protein is specific to foods with a particular taste profile. These results build upon the knowledge provided by neuroimaging studies on the response of reward-driven eating behaviors to increases in dietary protein.

## Figures and Tables

**Table 1:** Test day meals.

Breakfast	% E Protein		Lunch	% E Protein	
	15% (g)	30% (g)		15% (g)	30% (g)
Ham and potato bake:			Ham sandwich:		
Red potatoes	275	135	Bread	70	70
Ham	35	185	Ham	35	185
Cheddar cheese	20	20	Cheddar cheese	20	20
Toast	70	70	Red potato wedges	275	135
Butter	12	5	Butter	12	5

**Table 2:** Snacks foods chosen for the operant responding task.

Snack Food	Women (*n*)	Men (*n*)
Mini Oreos	8	1
M&Ms	2	2
Skittles	1	4
ReesePs	0	3
Doritos	9	10
Chees-Its	0	0
Pretzels	1	0
Pringles	1	0

## References

[1] Paddon-Jones D., Westman E., Mattes R.D., Wolfe R.R., Astrup A., Westerterp-Plantenga M. (2008). Protein, weight management, and satiety. Am. J. Clin. Nutr..

[2] Alonso-Alonso M., Woods S.C., Pelchat M., Grigson P.S., Stice E., Farooqi S., Khoo C.S., Mattes R.D., Beauchamp G.K. (2015). Food reward system: Current perspectives and future research needs. Nutr. Rev..

[3] Epstein L.H., Carr K.A., Lin H., Fletcher K.D. (2011). Food reinforcement, energy intake, and macronutrient choice. Am. J. Clin. Nutr..

[4] Epstein L.H., Leddy J.J., Temple J.L., Faith M.S. (2007). Food reinforcement and eating: A multilevel analysis. Psychol. Bull..

[5] Casperson S.L., Johnson L., Roemmich J.N. (2017). The relative reinforcing value of sweet versus savory snack foods after consumption of sugar- or non-nutritive sweetened beverages. Appetite.

[6] Journel M., Chaumontet C., Darcel N., Fromentin G., Tome D. (2012). Brain responses to high-protein diets. Adv. Nutr..

[7] Min D.K., Tuor U.I., Koopmans H.S., Chelikani P.K. (2011). Changes in differential functional magnetic resonance signals in the rodent brain elicited by mixed-nutrient or protein-enriched meals. Gastroenterology.

[8] Leidy H.J., Lepping R.J., Savage C.R., Harris C.T. (2011). Neural responses to visual food stimuli after a normal vs. Higher protein breakfast in breakfast-skipping teens: A pilot FMRI study. Obesity.

[9] Leidy H.J., Ortinau L.C., Douglas S.M., Hoertel H.A. (2013). Beneficial effects of a higher-protein breakfast on the appetitive, hormonal, and neural signals controlling energy intake regulation in overweight/obese, “breakfast-skipping,” late-adolescent girls. Am. J. Clin. Nutr..

[10] Griffioen-Roose S., Smeets P.A., van den Heuvel E., Boesveldt S., Finlayson G., de Graaf C. (2014). Human protein status modulates brain reward responses to food cues. Am. J. Clin. Nutr..

[11] Frank S., Laharnar N., Kullmann S., Veit R., Canova C., Hegner Y.L., Fritsche A., Preissl H. (2010). Processing of food pictures: Influence of hunger, gender and calorie content. Brain Res..

[12] Sayer R.D., Amankwaah A.F., Tamer G.G., Chen N., Wright A.J., Tregellas J.R., Cornier M.A., Kareken D.A., Talavage T.M., McCrory M.A. (2016). Effects of dietary protein and fiber at breakfast on appetite, ad libitum energy intake at lunch, and neural responses to visual food stimuli in overweight adults. Nutrients.

[13] Gosby A.K., Conigrave A.D., Lau N.S., Iglesias M.A., Hall R.M., Jebb S.A., Brand-Miller J., Caterson I.D., Raubenheimer D., Simpson S.J. (2011). Testing protein leverage in lean humans: A randomised controlled experimental study. PLoS ONE.

